# Neuropharmacology of Neuropathic Pain: A Systematic Review

**DOI:** 10.7759/cureus.69028

**Published:** 2024-09-09

**Authors:** Muhammad Umar Mian, Mishal Afzal, Aqsa A Butt, Muniba Ijaz, Kashaf Khalil, Maryam Abbasi, Marhaba Fatima, Mariam Asif, Saad Nadeem, Shivangi Jha, Binay K Panjiyar

**Affiliations:** 1 Internal Medicine, Allama Iqbal Medical College, Lahore, PAK; 2 Internal Medicine, Jinnah Sindh Medical University, Karachi, PAK; 3 Medicine, Darul Sehat Hospital, Karachi, PAK; 4 Internal Medicine, People’s University of Medical and Health Sciences for Women-Nawabshah, Nawabshah, PAK; 5 Obstetrics and Gynaecology, Pramukh Swami Medical College, Bhaikaka University, Anand, IND; 6 Cardiology/Global Clinical Scholars Research Training, Harvard Medical School, Boston, USA

**Keywords:** analgesics, nerve pain, neuropathic pain, neuropharmacology, neurotransmitters, pain modulation, peripheral neuropathy, pharmacological treatments, prisma, receptors

## Abstract

Neuropathic pain, a debilitating condition, remains challenging to manage effectively. An insight into neuropharmacological mechanisms is critical for optimizing treatment strategies. This systematic review aims to evaluate the role of neuropharmacological agents based on their efficacy, involved neurotransmitters, and receptors. A manual literature search was undertaken in PubMed including Medline, Cochrane Library, Google Scholar, Plos One, Science Direct, and clinicaltrials.gov from 2013 until 2023. Out of the 13 included studies, seven evaluated the role of gabapentinoids. Two main drugs from this group, gabapentin and pregabalin, function by binding voltage-gated calcium channels, lowering neuronal hyperexcitability and pain signal transmission, thereby relieving neuropathic pain. Four of the pooled studies reported the use of tricyclic antidepressants (TCAs) including amitriptyline and nortriptyline which work by blocking the reuptake of norepinephrine and serotonin, their increased concentration is thought to be central to their analgesic effect. Three articles assessed the use of serotonin-norepinephrine reuptake inhibitors (SNRIs) and reported them as effective as the TCAs in managing neuropathic pain. They work by augmenting serotonin and norepinephrine. Three studies focused on the use of selective serotonin reuptake inhibitors (SSRIs), modulating their effect by increasing serotonin levels; however, they were reported as not a highly effective treatment option for neuropathic pain. One of the studies outlined the use of cannabinoids for neuropathic pain by binding to cannabinoid receptors with only mild adverse effects. It is concluded that gabapentinoids, TCAs, and SNRIs were reported as the most effective therapy for neuropathic pain; however, for trigeminal neuralgia, anticonvulsants like carbamazepine were considered the most effective. Opioids were considered second-line drugs for neuropathic pain as they come with adverse effects and a risk of dependence. Ongoing research is exploring novel drugs like ion channels and agents modulating pain pathways for neuropathic pain management. Our review hopes to inspire further research into patient stratification by their physiology, aiding quicker and more accurate management of neuropathic pain while minimizing inadvertent side effects.

## Introduction and background

Pathology affecting the nervous system can present long-term consequences in the form of pain, termed neuralgia, neuropathy, or neuropathic pain. Neuropathic pain was defined by the 2011 International Association for the Study of Pain as "pain caused by a lesion or disease of the somatosensory system" [1]. Neuropathic pain originates from a dysfunctional and maladaptive response of the nervous system to any form of “damage.” Nerve injury triggers a sequence of changes that lead to the degeneration and regeneration of damaged nerve endings, followed by sensitization phenomena occurring either at peripheral or central sites [2]. Not all neuropathic pains are alike. Neuropathic pain can vary in symptoms and its subsequent underlying mechanisms, even when caused by the same condition. Patient stratification, according to the underlying pathophysiology, can greatly enhance specific treatment modifications, which will ultimately lead to better patient outcomes [3]. Neuropathic pain often involves a combination of sensory loss (numbness) and sensory gain (allodynia), with the specific manifestations differing among individuals and conditions. These variations may indicate distinct pain mechanisms at play within each person, potentially offering insights into their treatment responses [4].

Neuropathic pain is typically classified based on the underlying cause of nerve injury. While there are numerous potential causes, some of the more common ones include diabetes, leading to painful diabetic neuropathy (PDN), shingles resulting in post-herpetic neuralgia (PHN), amputation causing stump and phantom limb pain, as well as neuropathic pain following surgery or trauma, stroke, spinal cord injury, trigeminal neuralgia, and HIV infection. In some cases, the cause remains unidentified [2].

People suffering from neuropathic pain often experience significant disability, especially after enduring moderate to severe pain for many years [5]. This leads to a significant decline in quality of life, loss of employment, and higher healthcare expenses [6]. Neuropathic pain poses significant treatment challenges, as only a small percentage of individuals experience meaningful benefits from any single intervention. A multidisciplinary approach is now recommended, integrating pharmacological treatments with physical or cognitive therapies or both [2].

This study aims to understand the pharmacological agents that improve neuropathic pain and presents a systematic review of literature on a variety of drugs, including antidepressants, anticonvulsants, opioids, cannabis, and emerging treatments targeting specific receptors and neurotransmitters. It will try to give an updated answer to the question: “Which pharmacological agents play a role in improving neuropathic pain, and which neurotransmitters and receptors are involved?” We hope to encourage treatment particularization for the different patient strata by reviewing recent research data.

## Review

Methods

This systematic review follows the Preferred Reporting Items for Systematic Reviews and Meta-Analyses (PRISMA) guidelines to guarantee strict methodology.

Registration

This systematic review has been registered on PROSPERO, registration number CRD42024578582.

Databases and Search Strategy

**Figure 1:**
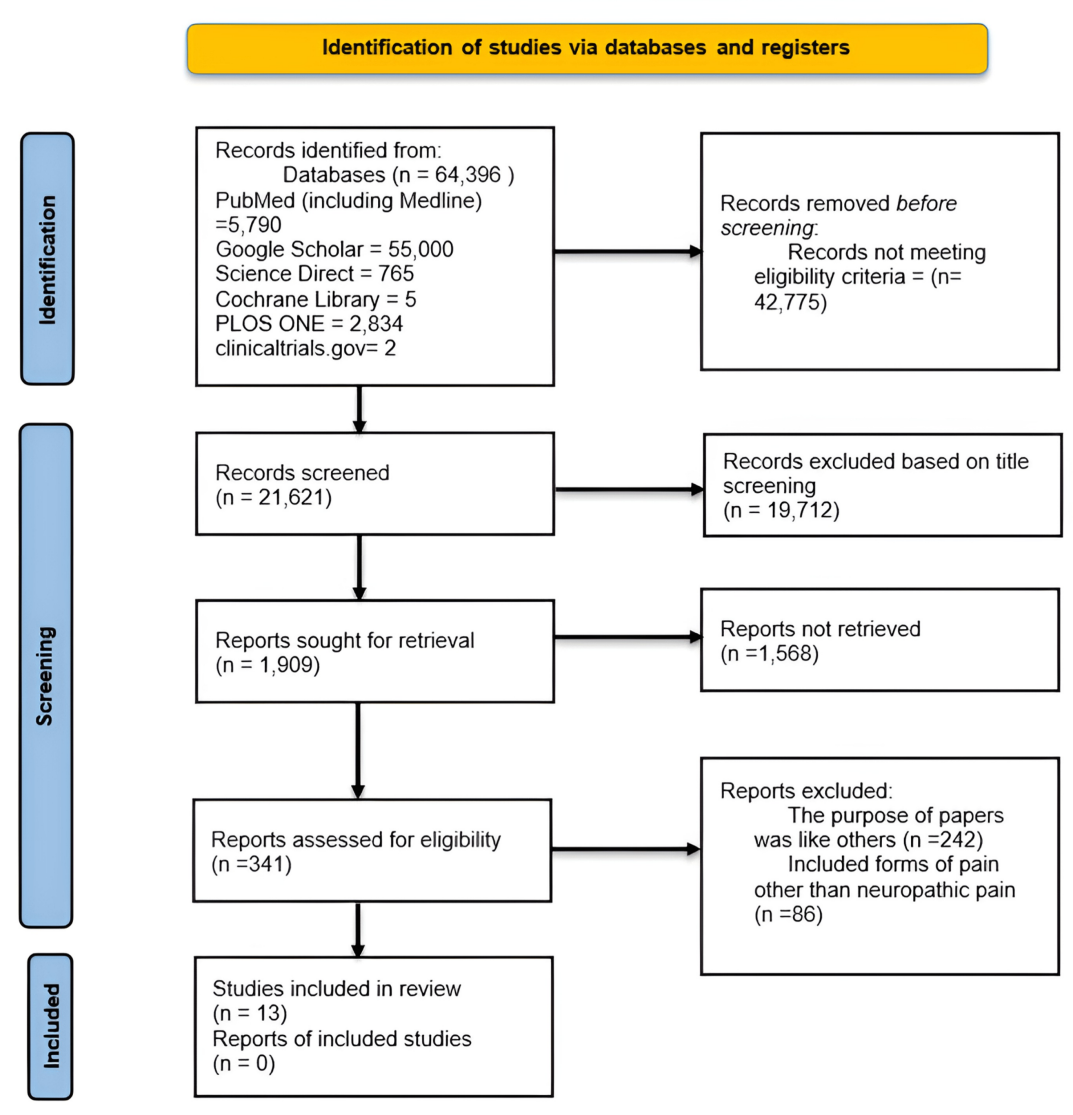
PRISMA flowchart of search strategy Six databases (Pubmed including Medline, Google Scholar, Science Direct, Cochrane Library, Plos One, and clinicaltrials.gov) were searched, screened, and shortlisted as per the Preferred Reporting Items for Systematic Reviews and Meta-Analyses (PRISMA) guidelines [7].

A manual literature search was conducted in PubMed including Medline, Cochrane Library, Google Scholar, Plos One, Science Direct, and clinicaltrials.gov from 2013 until 2023. The last search date was 31 December 2023. The complete search strategy and selection of studies are depicted in the PRISMA flowchart in Figure 1.

The databases and search methods are presented in Table 1 as follows:

**Table 1:** Search strategies of the databases searched along with the number of results before and after application of filters

Database	Search Strategy	Results before Filters	Filters Applied	Results after Filters
PubMed (Including MEDLINE)	(((pharmacology[MeSH Subheading]) OR (neuropathic pain[MeSH Subheading])) AND (("2013/11/20"[Date - MeSH] : "3000"[Date - MeSH]))) AND (neuropathic pain[Title/Abstract])	5790 (2023/11/20)	Human Studies; Free full text; ARTICLE TYPE: •Clinical Trial •Meta-Analysis •Randomized Controlled Trial •Systematic Review ; PUBLICATION DATE: 2013/05/02 - 2023/11/20 ; SPECIES: Humans ; ARTICLE LANGUAGE: English ; SEX: • Female • Male ; AGE: Adult: >18 years ; OTHER: MEDLINE	134
Google Scholar	Neuropharmacology of Neuropathic Pain	55,000 (2023/11/29)	From 2013 to 2023	19,200 Analyzed first 20 pages only.
Cochrane Central Register for Controlled Trials (Including Embase)	Neuropharmacology of Neuropathic Pain	5 (2023/11/29)	From 2013 to 2023 English only	3
Plos One	Neuropharmacology of Neuropathic Pain	2,834 (2023/12/31)	2013/05/02 - 2023/11/20	2,203
Science Direct	Neuropharmacology of Neuropathic Pain	765 (2023/12/15)	Open Access & Open Archives 2013 to 2023	79
Clinical Trials.gov	Condition: Neuropathic Pain Other terms: Chronic Pain Intervention: Neuropharmacology	2 (2023/12/15)	2013/05/02 - 2023/11/20	2

Eligibility Criteria

Studies were screened based on their respective types: randomized controlled trials (RCTs), systematic reviews, meta-analyses, and observational studies. The inclusion criteria included studies conducted on human adults, published in the last 10 years (2013 to 2023), and written in English. In contrast, non-English studies, studies with unavailable full texts or incomplete outcome data, studies involving pediatric populations, and studies published before 2013 were excluded. A detailed description of the inclusion and exclusion criteria is given in Table 2.

**Table 2:** Complete inclusion and exclusion criteria

	Inclusion Criteria	Exclusion Criteria
a)	Human Studies	Animal Studies
b)	From 2013 to 2023	Before 2013
c)	English Texts	Non-English Texts
d)	Gender: All	
e)	Age: >18 years old	Age: ≤ 18 years old
f)	Free Papers	Papers that needed to be purchased

Quality Appraisal

We used standardized quality assessment techniques to rigorously evaluate the quality of 13 selected studies. The Cochrane Risk of Bias tool for randomized controlled trials (RCTs), the SANRA (Scale for the Assessment of Non-Systematic Review Articles) tool for observational studies, and the AMSTAR (A Measurement Tool to Assess Systematic Reviews) checklist for systematic reviews were used to evaluate the quality of the studies as elucidated in Table 3 below. Three reviewers independently conducted the quality assessment. Any and all disputes were resolved through mutual discussion. A fourth reviewer was consulted if the need arose.

**Table 3:** Quality assessment tools used AMSTAR: A Measurement Tool to Assess Systematic Reviews; SANRA: Scale for the Assessment of Narrative Review Articles.

Quality Assessment Tool	Study Type
Cochrane Risk of Bias Assessment Tool [8]	Randomized Controlled Trials
AMSTAR Checklist [9]	Systematic Reviews and Meta-Analysis
SANRA Checklist [10]	Any other without a Clear methods section

**Figure 2:**
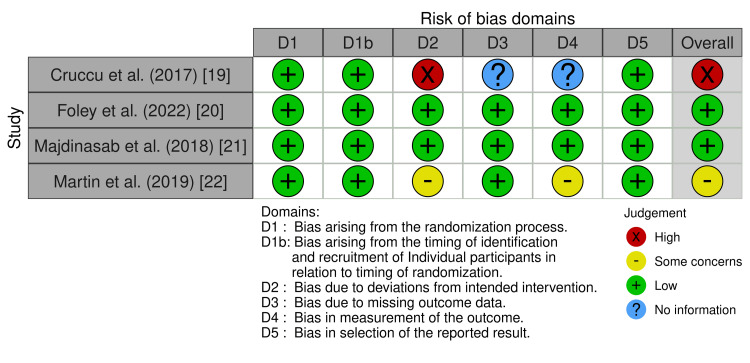
Cochrane cluster randomized trials risk of bias assessment Cochrane risk of bias tool was implemented for randomized controlled trial studies [8]. One study had a high risk, two studies had a low risk, and one study had some concerns about the risk of bias.

Figure 2, Table 4, and Table 5 present the specific overall scores for risks of bias for every study that was chosen.

**Table 4:** AMSTAR quality assessment tool for systematic reviews PICO: Population/Patient Intervention Comparison Outcome; AMSTAR: A Measurement Tool to Assess Systematic Reviews [9].

AMSTAR				
	Wiffen et al. (2013) [16]	Chen et al. (2022) [17]	Wiffen et al. (2016) [2]	Bennici et al. (2021) [18]
Did the research questions and inclusion criteria for the review include the components of PICO?	Yes	Yes	No	Yes
Did the report of the review contain an explicit statement that the review methods were established prior to the conduct of the review and did the report justify any significant deviations from the protocol?	Partial Yes	Yes	Partial Yes	Partial Yes
Did the review authors explain their selection of the study designs for inclusion in the review?	Yes	Yes	Yes	Yes
Did the review authors use a comprehensive literature search strategy?	Yes	Yes	Partial Yes	Partial Yes
Did the review authors perform study selection in duplicate?	Yes	Yes	Yes	Yes
Did the review authors perform data extraction in duplicate?	Yes	Yes	No	No
Did the review authors provide a list of excluded studies and justify the exclusions?	No	No	Yes	Yes
Did the review authors describe the included studies in adequate detail?	Yes	No	No	Yes
Did the review authors use a satisfactory technique for assessing the risk of bias (RoB) in individual studies that were included in the review?	Yes	Yes	No	Yes
Did the review authors report on the sources of funding for the studies included in the review?	No	No	Yes	No
If meta-analysis was performed did the review authors use appropriate methods for statistical combination of results?	No	Yes	No	No
If meta-analysis was performed, did the review authors assess the potential impact of RoB in individual studies on the results of the meta-analysis or other evidence synthesis?	No	Yes	No	No
Did the review authors account for RoB in individual studies when interpreting/ discussing the results of the review?	Yes	Yes	Yes	Yes
Did the review authors provide a satisfactory explanation for, and discussion of, any heterogeneity observed in the results of the review?	Yes	Yes	No	Yes
If they performed quantitative synthesis did the review authors carry out an adequate investigation of publication bias (small study bias) and discuss its likely impact on the results of the review?	Yes	Yes	No	No
Did the review authors report any potential sources of conflict of interest, including any funding they received for conducting the review?	Yes	Yes	Yes	No

**Table 5:** SANRA quality assessment tool for articles without a clear methods section SANRA= Scale for the Assessment of Non-Systematic Review Articles [10].

SANRA					
	Fornasari et al. (2017) [11]	Kersten et al. (2015) [12]	Obata et al. (2017) [13]	Alles et al. (2018) [14]	Gambeta et al. (2020) [15]
Justification of the article's importance for the readership	2	2	2	2	2
Statement of concrete aims or formulation of questions	2	2	2	2	2
Description of the literature search	0	0	2	0	0
Referencing	2	2	2	2	2
Scientific reasoning	2	1	2	1	2
Appropriate presentation of data	1	2	2	2	2
Total Score	9	9	12	9	10

Results

Three authors worked on the data extraction and compilation of subsequent results. We extracted data from the 13 studies included in our article. The results are presented in Table 6 and discussed below.

**Table 6:** Key findings and characteristics of all included studies

Study Title	Study Design	Pharmacological Agents	Neurotransmitters	Receptors	Participants Included	Key Findings
Alles and Smith (2018) [14]	Narrative Review	Gabapentinoids, Opioids, TCAs, SNRIs, Sodium channel blockers, Potassium channel openers, Calcium channel blockers, Hyperpolarization-Activated Cyclic Nucleotide–Gated Channels Modulators, Transient Receptor Potential (TRP) Channel Modulators, Adenosine A3 Receptor Agonists, Colony-Stimulating Factor 1 Inhibitors, Purinergic Ionotropic 2X4 (P2X4) Receptor Antagonists, Brain-Derived Neurotrophic Factor (BDNF) Pathway Modulators	Norepinephrine, Serotonin, Glutamate, Substance P	α2δ-1 subunit of voltage-gated Ca2+ channels, Kv7.2 and Kv7.3 subunits, TRPV, Adenosine A31, P2X4	Not Specified	Gabapentinoids are first-line treatments; antidepressants and opioids are also used but with varying efficacy. Research is exploring new drug targets including specific ion channels, receptors (TRPV1, adenosine A3, P2X4), and pathways involved in pain modulation.
Chen and Li (2022) [17]	Network Meta-Analysis and Systematic Review	Gabapentin, pregabalin, fluoxetine, lamotrigine, duloxetine, sertraline, amitriptyline, carbamazepine, vitamin B	Adrenaline, 5-hydroxytryptamine (5-HT), gamma-aminobutyric acid (GABA), glutamate	Not specified	1040	Gabapentin and pregabalin showed the most significant effects in relieving central poststroke pain. [Gabapentin > pregabalin > fluoxetine > lamotrigine > duloxetine > serqulin > amitriptyline > carbamazepine > vitamin B] However, study limitations, including small sample size and lack of proper RCTs, warrant further investigation.
Cruccu et al. (2018) [19]	Randomized Controlled Trial	Capsaicin 8% patch, pregabalin	Not specified	Low-threshold, mechano-sensitive C fibers (C tactile fibers). Capsaicin is a potent, highly selective vanilloid receptor subtype 1 (TRPV1) agonist	488	Capsaicin 8% patch was superior to pregabalin in relieving dynamic mechanical allodynia and associated with fewer adverse events.
Foley et al. (2022) [20]	Randomized Controlled Trial	Fluoxetine, Riluzole, Amiloride	Glutamatergic neurotransmission depression	Central receptors: Riluzole: Na- Channel inactivator Amiloride: Blocks acid-sensing ion channels (a mechanism of pain) in CNS (Central Nervous System) + PNS (Peripheral Nervous System)	445	No statistically significant benefit in neuropathic pain symptoms was identified in participants receiving fluoxetine, riluzole, or amiloride compared to placebo.
Fornasari D (2017) [11]	Narrative Review	TCAs, SNRIs, gabapentinoids, lidocaine, opioids	Serotonin, norepinephrine (NE)	Alpha-1 adrenergic receptors, NMDA receptors, calcium channels, Na-Channels, seven transmembrane G protein-coupled receptors, which are divided into three classes: mu, delta, and kappa receptors. Coupling of opioid receptors to calcium and potassium channels is thought to be a central mechanism of analgesia.	Not specified	Antidepressants and anticonvulsants, particularly TCAs and gabapentinoids, are effective first-line treatments for neuropathic pain. Opioids are effective but carry risks of abuse and addiction. Tapentadol is a new class of dual opioid analgesics, which inhibits noradrenaline uptake as well as acts as an agonist at mu opioid receptors.
Gambeta et al. (2020) [15]	Narrative Review	Carbamazepine, Oxcarbazepine, Gabapentin, Pregabalin, Lamotrigine, Phenytoin, Baclofen, Botulinum toxin type A, Eslicarbazepine	Glutamate,	Voltage-gated sodium channels	Not Specified	The first-line treatment for trigeminal neuralgia is pharmacological, with carbamazepine and oxcarbazepine being the most commonly prescribed drugs. These medications work by modulating voltage-gated sodium channels and reducing neuronal excitability. Other pharmacological agents, such as gabapentin, pregabalin, and botulinum toxin type A, can also be used. Surgical options are available for patients who do not respond to medication or experience intolerable side effects.
Kersten et al. (2015) [12]	Case Series	Cetuximab	Not specified	EGFR, MAPK, human epidermal growth factor receptor (HER), Na-Channels	20	EGFR inhibition with cetuximab effectively relieved neuropathic pain with minimal adverse events.
Majdinasab et al. (2019) [21]	Randomized Controlled Trial	Duloxetine, gabapentin	Serotonin, norepinephrine, GABA	Not specified	104	Both duloxetine and gabapentin significantly improved pain scores over time, with duloxetine associated with fewer side effects.
Martin et al. (2019) [22]	Randomized Controlled Trial	Ketamine, Dextromethorphan, memantine	Not specified	NMDA receptors	60	Oral dextromethorphan temporarily extended ketamine pain relief and improved cognitive-affective dimensions in neuropathic pain patients.
Bennici et al. (2021) [18]	Systematic Review	Tetrahydrocannabinol (THC), Cannabidiol (CBD)	Dopamine	CB1 & CB2 (Cannabinoid receptor 1 & 2)	1080	Cannabis (mainly a combination of THC and CBD) caused reduction in pain intensity and spasticity in patients with neuropathic pain due to fibromyalgia, radiculopathy, chronic non-cancerous pain, spinal cord cancer pain, diabetic neuropathy (DN), complex regional pain syndrome, pelvic neuropathic pain, spinal cord injury pain, multiple sclerosis, vertebral artery occlusion, arachnoid cysts, syringomyelia, and HIV-associated distal sensory predominant polyneuropathy.. Dizziness, headache, nausea, mild neurocognitive impairment, sedation, and “drug high” are common, while severe adverse events are rare.
Obata H (2017) [13]	Narrative Review	TCAs, SNRIs	Noradrenaline, serotonin (5-HT)	Alpha-2 adrenergic receptors	Not specified	Antidepressants inhibit neuropathic pain by increasing noradrenaline in the spinal cord and acting on the locus coeruleus. TCAs have stronger analgesic effects than drugs that selectively inhibit reuptake of only one neurotransmitter.
Wiffen et al. (2013) [16]	Systematic Review	Gabapentin, pregabalin, carbamazepine, lamotrigine, oxcarbazepine, topiramate, lacosamide, clonazepam, phenytoin, valproic acid	Not specified	Alpha-2 delta calcium channels, sodium channels, NMDA receptors	Not specified	Gabapentin and pregabalin showed reasonably good evidence for efficacy in painful diabetic neuropathy and postherpetic neuralgia, with a modest effect size. Other antiepileptic drugs had mixed or inconclusive evidence for neuropathic pain.
Wiffen et al. (2016) [2]	Systematic Review	Paracetamol/acetaminophen	Not specified	Central receptors	Not specified	No studies meeting inclusion criteria; evidence supporting the use of paracetamol for neuropathic pain is lacking.

Gabapentinoids

Seven of the included studies evaluated the role of gabapentinoids in managing neuropathic pain [11,14-17,19,21]. They are considered the first-line treatments for various conditions causing neuropathic pain. Gabapentin and pregabalin, the two main drugs in this class, have shown efficacy in relieving central poststroke pain, painful diabetic neuropathy, and postherpetic neuralgia [17]. Their effects are modulated via binding of the α2δ-1 subunit of voltage-gated calcium channels, distributed throughout the nervous system. Their binding causes the release of excitatory neurotransmitters, including glutamate, substance P, and norepinephrine, reducing neuronal hyperexcitability and pain signal transmission [21].

*Tricyclic Antidepressants* (*TCAs)*

Four of the pooled studies examined the use of TCAs for treating neuropathic pain [11,13,14,17]. Of these studies, three identified TCAs as a first-line treatment [11,13,17], while one suggested they may come as a secondary choice given their potential side effects [14]. Among TCAs, amitriptyline and nortriptyline are the most extensively studied antidepressants for neuropathic pain. These medications have demonstrated efficacy in treating various neuropathic pain conditions, including peripheral neuropathy, postherpetic neuralgia, and pain following spinal cord injury. TCAs work primarily by blocking the reuptake of norepinephrine and serotonin in the central nervous system. The increase in noradrenaline, in particular, is thought to be central to their analgesic effects. Despite their effectiveness, TCAs are associated with potential side effects. Nortriptyline is often preferred over amitriptyline due to its milder side effect profile [13].

*Serotonin-Norepinephrine Reuptake Inhibitors​​​​​​​* (*SNRIs)*

Four articles reported the use of SNRIs for neuropathic pain [11,13,14,21]. Venlafaxine, duloxetine, and other SNRIs are antidepressants that have been used for neuropathic pain, and they perform the same as TCAs. Majdinasab et al. showed that they are effective by elevating the concentrations of serotonin and norepinephrine in the central nervous system, which are involved in pain regulation [21]. Alles and Smith have described SNRIs as being among the first-line management options in the same way as TCAs [14]. However, Obata H pointed out that even though SNRIs are effective, they might not be as effective as TCAs in the treatment of neuropathic pain, but they are better tolerated than the latter [13].

Opioids

Two studies report the effects of opioids on neuropathic pain [11,14]. Endogenous opioids work by using the mu, delta, and kappa receptors, which subsequently upregulate the inhibitor G protein, thus inhibiting the adenylate cyclase along with the cAMP. However, mechanisms common to both endogenous and exogenous opioids include their coupling to the calcium and potassium channels, contributing to their role as central analgesics. However, Fornasari et al. have tagged opioids as second and third-line agents when it comes to neuropathic pain. A major factor for this nomination is their poor side effect profile, including but not limited to addiction, diversion, and abuse potential [11]. Alles and Smith have categorized opioids as second-line agents in neuropathic pain due to the loss of u-opioid receptors in any kind of nerve injury [14]. Furthermore, they have extended the debate to include that any form of pain that is resistant to opioid use is likely neuropathic in nature due to the same mechanism [14].

Anticonvulsants

Besides gabapentinoids, other anticonvulsants like carbamazepine and oxcarbazepine play a crucial role in managing neuropathic pain, particularly trigeminal neuralgia [15]. Three of the included studies reported findings on anticonvulsants [15-17]. These medications primarily act by modulating voltage-gated sodium channels, reducing the excessive firing of neurons associated with this condition. By stabilizing neuronal membranes and inhibiting repetitive action potentials, they effectively alleviate pain. However, potential side effects include somnolence, dizziness, and drowsiness [16,17].

*Selective Serotonin Reuptake Inhibitors​​​​​​​* (*SSRIs)*

Three articles focused on the application of SSRIs for neuropathic pain, among which fluoxetine was the most common [11,13,20]. These drugs are known to help by increasing serotonin levels in the brain. Foley et al. demonstrated that while serotonin is widely associated with mood regulation since it is a neurotransmitter, it also influences pain transmission by altering pain channels, possibly resulting in the relief of pain [20]. However, Obata et al. indicated that often, SSRIs are not regarded as a very effective modality in managing chronic neuropathic pain [13].

Cannabinoids

Bennici et al. mention the use of cannabis and cannabinoids for neuropathic pain relief, the review mentions that nabiximoles, products of cannabidiol and delta-9-tetrahydrocannabinol (THC), are approved for neuropathic pain and spasticity due to multiple sclerosis in certain regions [18]. Cannabinoids (CB) work by activating two receptors within the endocannabinoid system: cannabinoid receptor type 1 (CB1) and type 2 (CB2). The endocannabinoid system (eCS) is a biological network comprising endocannabinoids-endogenous lipid-based retrograde neurotransmitters that bind to cannabinoid receptors and cannabinoid receptor proteins distributed throughout the vertebrate central nervous system. Common side effects include dizziness, headache, nausea, mild neurocognitive impairment, sedation, and a "drug high," while severe adverse events are rare.

*N-Methyl-D-Aspartate* ​(*NMDA) Receptor Blockers*

Martin et al. reported that Ketamine is effective for postoperative pain, post-herpetic neuralgia, and phantom limb pain [22]. However, due to its poor side effect profile, including hepatic, cardiovascular, and psychodysleptic side effects, other NMDA receptor blockers like dextromethorphan and memantine can be used in its place to prolong ketamine’s analgesic effects [22]. Another benefit of these alternative options is that they can be administered orally, as opposed to the intravenous route of ketamine, improving medication compliance [22].

Paracetamol

Wiffen et al. have reported that paracetamol, with or without codeine or hydrocodone, is one of the first-line drugs used in postherpetic neuralgia, painful diabetic neuropathy, neuropathic low back pain, and phantom limb pain. The mechanism of paracetamol is still a point of debate, but it is said to be working by inhibiting the cyclooxygenase (COX) receptors in the brain, spleen, and lungs. However, there is no high-quality evidence supporting or contesting paracetamol’s efficacy in reducing neuropathic pain [2].

Emerging Therapies

Ongoing research is exploring novel drug targets for neuropathic pain management. These include specific ion channels, such as transient receptor potential vanilloid-1 (TRPV-1) and P2X purinoceptor 4 (P2X4), as well as receptors like adenosine A3. Additionally, pathways involved in pain modulation, such as glial cell activation and inflammatory processes, are being investigated as potential therapeutic targets [14]. Kersten et al. showed that any neurotransmitter that inhibits the EGFR (Epidermal Growth Factor Receptor) system blocks the MAPK (mitogen-activated protein kinases) pathway, eventually resulting in the inhibition of neuropathic pain. Another proposed mechanism of the EGFR-inhibitor drugs is their effect on the sodium channels, as demonstrated by improved outcomes when used alongside gabapentin and pregabalin in tumor cell lines [12].

Discussion

Neuropathic pain is challenging to manage and significantly impacts a patient’s quality of life [23]. Understanding the involvement of receptors and neurotransmitters can help tailor a regimen specific to the patient. Neuropathic pain can be central or peripheral, but regardless of the origin, gabapentinoids, TCAs, and SNRIs are the recommended first-line therapy [24]. However, conditions like trigeminal neuralgia, a type of peripheral neuropathic pain, do not respond well to these treatments and require carbamazepine or oxcarbazepine as a specific, effective therapy [25]. Similarly, opioids like tramadol and tapentadol, are the recommended second-line therapy [24]; however, the first line in intermittent exacerbation of neuropathic pain and cancer-related pain [26]. The National Institute for Health and Care (NICE) recommends that for managing neuropathic pain pharmacologically, patients should be offered a choice among amitriptyline, duloxetine, gabapentin, or pregabalin as initial treatment options (excluding trigeminal neuralgia). If the first, second, or third medications are ineffective or poorly tolerated, it is recommended to switch the medication [2]. This difference could be because of variations in underlying disease pathophysiology, differences in nerve involvement, and variability in pain receptors and neurotransmitters involved.

Antidepressants

Antidepressants exert their pharmacological effect by binding to noradrenaline and serotonin receptors, leading to increased levels of serotonin and norepinephrine in the synaptic cleft. According to a review by Obata et al., antidepressants that inhibit the reuptake of both neurotransmitters have stronger analgesic effects than a drug that selectively inhibits only one neurotransmitter. Additionally, norepinephrine plays a greater role than serotonin in the analgesic effect, and the number needed to treat (NNT) for SSRIs is higher than that for TCAs and SNRIs [13]. The exact mechanism of neuropathic analgesia of antidepressants is still unknown and requires further high-quality research. One proposed mechanism is that these drugs act by exerting their effects on the norepinephrine and serotonin neurotransmitters in the spinal pain pathway. Additional proposed mechanisms involve the modulation of histamine and sodium channels [27]. P2X4 is an adenosine 5’-triphosphate (ATP) cation channel receptor that plays a role in neuroinflammation. So far, duloxetine is one drug that has been shown to inhibit these receptors, thereby improving neuropathic pain. However, more in-depth research is yet to be seen [28].

Anticonvulsants

Anticonvulsants such as pregabalin and gabapentin bind to alpha-2-delta calcium channels. Gabapentinoids decrease the calcium channel currents by influencing the number of available calcium channels in the plasma membrane [29]. These drugs bind the pre-synaptic calcium channels that modulate the release of excitatory neurotransmitters by blocking calcium entry [30]. Gabapentin has been shown to reduce presynaptic gamma-aminobutyric acid (GABA) release in the locus coeruleus via the GABA-B receptors, which subsequently increases the glutamate neurotransmitter [31]. Between gabapentin and pregabalin, the latter has a bioavailability of more than 90% in contrast to the 30-60% of gabapentin. In addition, pregabalin has a linear dose-response relationship as it is absorbed in the small intestine as well as the proximal colon as compared to only small intestinal absorption of gabapentin. Admittedly, the absorption of pregabalin significantly decreases by food intake, but that of gabapentin does not [32]. This difference in pharmacokinetics could be of potential importance to patients with small intestinal absorptive or transit conditions. Active Crohn’s disease, radiation enteritis, hyperthyroidism, and neuroendocrine tumors decrease the small intestinal transit time, thereby potentially decreasing gabapentin absorption [33]. However, purely malabsorptive conditions like celiac disease do not alter pregabalin absorption [34]. Lamotrigine has shown efficacy as an adjunct therapy to TCAs, SNRIs, and gabapentinoids for spinal cord injury-induced neuropathic pain and intractable trigeminal neuralgia [35,36].

Topical Lidocaine

Topical lidocaine is considered a second-line agent and blocks voltage-gated sodium channels when administered as a patch [37], with mild local reactions as side effects [38]. Notedly, topical anesthetics should be distinguished from topical patches, as the latter have major systemic absorption as their primary mechanism, while the former only exert their effects close to the site of injection. Lidocaine can provide local anesthetic effects in the form of medicated plasters, gels, and sprays [39]. Topical lidocaine can be used as an adjunct in opioid-sparing pain management as it has been shown to be beneficial in post-surgical persistent pain (PSPP), PHN, diabetic peripheral neuropathy (DPN), carpal tunnel syndrome (CTS), chronic lower back pain (CLBP), and osteoarthritis (OA) [40].

Opioids

Strong opioids like morphine, oxycodone, and hydromorphone, as well as weaker opioids such as tramadol, have shown efficacy in treating neuropathic pain, comparable to antidepressants in terms of the NNT. Despite their effectiveness, opioids are typically considered second or even third-line treatments due to the risk of adverse reactions, potential for abuse, diversion, and addiction [41]. The analgesic effects of opioids arise from their actions in the brain, brainstem, spinal cord, and, under certain conditions, the peripheral terminals of primary afferent neurons. Endogenous opioid peptides, including β-endorphin, enkephalins, and dynorphins, bind to G protein-coupled receptors, classified into mu, delta, and kappa receptors. These receptors are linked to inhibitory G proteins, and their activation inhibits adenylate cyclase and intracellular cAMP production. A key mechanism of opioid-induced analgesia involves the coupling of these receptors to calcium and potassium channels [42].

Within the dorsal horn of the spinal cord, mu receptors predominate, with over 70% located pre-synaptically at the central terminals of nociceptors (C and A delta fibers). The remaining 30% are found post-synaptically on dendrites of second-order spinothalamic neurons and interneurons [42]. These interneurons are primarily responsible for releasing endogenous opioids like beta-enkephalin and endorphins, which act on mu receptors in the dorsal horn. Interneuron activation depends on descending pathway activity or direct stimulation by descending fibers. Activation of presynaptic mu receptors inhibits calcium ion channels, preventing neurotransmitter release, while activation of postsynaptic mu receptors opens potassium ion channels, causing potassium efflux and hyperpolarization of the projecting cell. Thus, stimulating mu-opioid receptors in the spinal cord effectively blocks synaptic transmission, reducing nociceptive stimuli reaching the thalamus and cortex, where pain is consciously perceived [42]. Despite their efficacy, the long-term use of opioids for nonmalignant pain remains controversial due to concerns about the potential development of tolerance to their analgesic effects, and the risk of addiction [42].

Riluzole and Amiloride

Riluzole is known to inactivate voltage-dependent sodium channels and depress glutamatergic neurotransmission, offering another potential avenue for pain management. Amiloride, which blocks acid-sensing ion channels implicated in both central and peripheral pain mechanisms, is used in the treatment of migraines. These insights into various drug mechanisms emphasize the need for continued research to enhance our understanding and treatment of neuropathic pain [20].

Riluzole was initially reported to be of little to no use for treating peripheral neuropathic pain [43]. However, recent studies have shown improved outcomes for patients experiencing cervical myelopathy who were treated with riluzole versus placebo [44]. Riluzole provides benefits to patients with Alzheimer’s disease by preserving cerebral glucose metabolism and to ALS patients by improving survival. It has also shown benefits to patients with spinal cord injury (SCI) in decreasing their pain [45,46].

Amiloride is a sodium channel-blocking diuretic that is commonly used for congestive heart failure and hypertension. One of its mechanisms of action is to inhibit the acid-sensing ion channels (ASIC). This effect has been shown to improve neuropathic pain by adopting the intrathecal injection approach for acute and chronic pains [47].

Vitamin B Complex

The Vitamin B complex including thiamine (B1), pyridoxine (B6), folic acid (B9), and cyanocobalamin (B12) have shown improvements in neuropathic pains of various etiologies, specifically diabetic neuropathy. These vitamins have the added advantage of being neuroprotectors of the entire nervous system, where B1 has a site-directed antioxidant property, B6 modulates nerve metabolism, and B12 stabilizes the myelin sheaths, blocks sensory nerve conduction, and decreases ectopic nerve firing. It is worth noting that B12 has a special role in nerve-regenerating activities as well. It has been shown to not only improve neuropathic pain symptoms but also completely regenerate and recover lost myelin sheaths and physiological nerve conduction velocities [48-51]. B12 is of particular importance for diabetic patients who are taking metformin, as this drug has been shown to decrease B12 absorption in the body, contributing to increased neuropathic pain [52].

Cannabinoids

Cannabinoids work by activating the cannabinoid receptors CB1 and CB2. The cannabinoid receptors work by blocking the voltage-gated calcium channels, inhibiting adenylate cyclase, and decreasing the cAMP levels [53]. Cannabinoids have been shown to have efficacy for improving neuropathic pain but only for the short term. This is due to their shorter efficacy window as well as a multitude of side effects, including neurological and psychiatric disorder development with long-term use [54]. Limitations to cannabis use for neuropathic pain also include a risk of dependence leading to cannabis use disorder. In addition to dependence, long-term use can also increase the risk of withdrawal symptoms once therapy is ceased, including but not limited to symptoms of anger, aggression, decreased appetite, irritability, restlessness, and insomnia [55].

Capsaicin

Capsaicin, a natural spice, has been historically used for its topical anesthetic effects. It exerts its effects through the activation of the transient receptor potential vanilloid-1 (TRPV1) on nerve fibers. Capsaicin use avoids the systemic side effects of systemic drugs and works by inducing a momentary discomfort at the application area, followed by a prolonged anesthetic response [56]. Capsaicin has primarily shown efficacy in treating postherpetic neuropathic pain and HIV neuropathy. The main adverse effects are transient pain and erythema over the application site [57].

Paracetamol

Paracetamol is one of the most commonly used analgesics and antipyretics. It is postulated to work by the inhibition of the central COX pathway, leading to an enhanced descending serotonergic effect. However, it is distinct from non-steroidal anti-inflammatory drugs (NSAIDs) as it does not have any peripheral anti-inflammatory effects [58]. Paracetamol is the first-line therapy for patients with OA [59]. OA has been shown to have a neuropathic pain component, as evidenced by a pathological innervation of the degenerative cartilage in the joint space [60]. Furthermore, the efficacy of paracetamol is being debated for OA, especially for long-term use. However, it is postulated that paracetamol has good synergistic actions when used as a combination therapy for neuropathic pain [61]. Paracetamol has shown considerable efficacy when used in combination with opioids for rheumatoid arthritis (RA), OA, and diabetic neuropathy [58].

Neuropathic pain has multiple etiologies, and it stands to reason that the identification of each of these underlying pathologies and their mechanisms can aid in improved outcomes for this condition. However, drug side effects may also depend on the etiology, for example, drugs with CNS-related side effects may be less tolerated in patients with CNS lesions [41]. Majdinasab et al. presented a proposal for painful diabetic peripheral polyneuropathy (PDPP). The proposal suggested that other pain-controlling medications, such as TCAs or gabapentin, should be used in the first week. After one week, these medications should be discontinued, and only duloxetine should be used [21], considering that gabapentin works fast, and duloxetine works late but shows lesser side effects. Although this proposal needs more investigations and further studies, it hints at the importance of combined therapy.

Limitations

Our systematic literature review has limitations. We only included studies that provided free, full text, and paid or closed-access articles were excluded. Our study only includes English-based journals/articles, and this can be perceived as a major limitation as a lot of great research exists in the non-English literature and language, just like any other parameter should not be a barrier towards scientific advancement. Neuropathic pain has several manifestations, and though we tried to include the most common ones, there are still other forms and etiologies of it that were not discussed in this article. Similarly, we tried to include the most common drugs used for neuropathic pain, as well as novel and less common therapies, yet there may be several other therapies that were not discussed. Evidence of efficacy for drugs like paracetamol was lacking in any high-quality information, and evidence for the emerging therapies might still be in their early stages. Further high-quality research is awaited on many of this condition's avenues.

## Conclusions

Neuropathic pain can be caused by a number of underlying conditions. It can present in a variety of symptoms, like numbness, tingling, creeping sensations, sharp, shooting pains, lancinating pains, allodynia, hyperalgesia, hypersensitivity to touch, and burning sensations. Neuropathic pain is usually considered chronic in nature. A big part of this might be due to the fact that it is usually associated with chronic underlying conditions, like diabetes mellitus and HIV, and chronic central and peripheral nervous system diseases, like multiple sclerosis, spinal stenosis, spinal compression, and central post-stroke syndromes.

Regardless of the etiology, neuropathic pain, in general, can be managed on its own with specific treatment options. For decades, a ‘one-size-fits-all’ approach has been used for managing neuropathic pain. Advancements in science urge us to explore better avenues with greater precision to manage this chronic debilitating condition. Given the complex nature of neuropathic pain, future therapeutic strategies may benefit from drug combinations. While the development of new treatments for neuropathic pain faces significant challenges, a deeper understanding of the mechanisms involved offers hope for more effective and targeted therapies. This review is one step toward understanding the basic pathophysiology of each kind of neuropathic pain caused by different underlying etiologies, which may lead to highly customized and tailored regimens for each individual patient. We hope that this review paves the way for further research along these lines so that we can hope to minimize the detrimental effects of this condition on the quality of life of millions of patients.
